# Fluctuation of intraocular pressure in glaucoma patients before and after trabeculectomy with mitomycin C

**DOI:** 10.1371/journal.pone.0185246

**Published:** 2017-10-04

**Authors:** Joanna Wasielica-Poslednik, Julian Schmeisser, Esther M. Hoffmann, Veronika Weyer-Elberich, Katharina Bell, Katrin Lorenz, Norbert Pfeiffer

**Affiliations:** 1 Department of Ophthalmology, University Medical Center, Johannes Gutenberg- University, Mainz, Germany; 2 Institute for Medical Biostatistics, Epidemiology and Informatics (IMBEI), University Medical Center, Johannes Gutenberg University, Mainz, Germany; Bascom Palmer Eye Institute, UNITED STATES

## Abstract

**Purpose:**

Intraocular pressure (IOP) fluctuation is considered as a risk factor for glaucoma progression. We investigated IOP values and IOP fluctuation before and after trabeculectomy (TE) with mitomycin C (MMC) measured by 48-hour diurnal-nocturnal-IOP-profiles (DNP).

**Methods:**

Pre- and postoperative DNPs of 92 eyes undergoing primary TE with MMC were analysed. Each 48-hour IOP-profile involved 10 IOP measurements (8:00 a.m., 2:00 p.m., 6:00 p.m., and 9:00 p.m. in sitting and at 00:00 in supine position). The “preoperative DNP” was performed a few weeks before TE. The “postoperative DNP” was performed at least six months (range: 6 months—2 years) after TE. Mean IOP values and IOP fluctuations were calculated.

**Results:**

After TE with MMC mean IOP was reduced from 16.94±3.83 to 11.26±3.77 mmHg at daytime and from 18.17±4.26 to 11.76±3.90 mmHg at night. At daytime mean IOP-fluctuation decreased from 8.61±4.19 to 4.92±2.52 mmHg, at night from 3.15±2.95 to 1.99±1.82 mmHg. Mean IOP was lower on the second day of the preoperative DNP. This effect was not present in the postoperative DNP. Preoperatively, IOP was controlled in all eyes with a mean of 3.22±0.94 antiglaucomatous agents. Postoperatively, IOP≤15 mmHg was achieved in 71.7%, IOP≤18 mmHg in 77.1% and a decrease in IOP of >30% in 47.8% without antiglaucomatous therapy. Postoperatively, pseudophakia was associated by a higher mean IOP-fluctuation compared to the phakic eyes.

**Conclusions:**

TE with MMC significantly reduces both mean IOP-values and IOP- fluctuations at day and night at least 6 months postoperatively. The effect of TE on the IOP fluctuation was less pronounced in pseudophakic eyes.

## Introduction

Reducing intraocular pressure (IOP) is the only evidence-based intervention that can delay the onset and progression of glaucoma, which is sight threatening progressive optic neuropathy [1–2]. A major risk factor for glaucoma is an elevated IOP which is specified as values measured above 21 mmHg. Nevertheless, we know that rather than having an absolute high IOP above 21 mmHg, many patients suffer from an individual high IOP presenting with normal IOP ranges accompanied by glaucomatous damage and disease progression. Especially IOP-fluctuations, defined as the difference between maximal and minimal IOP values measured during a day, are relevant for progression in glaucoma damage [3–6]. Daily IOP-fluctuations ranging from 3.17 to 5 mmHg are considered to be physiological [7]. In contrast, glaucoma patients can show larger IOP variations ranging from 4.8 to 11 mmHg. According to other studies, IOP-fluctuation was not an independent risk factor of glaucoma or glaucoma progression [8–10]. Evaluation of IOP-values and their diurnal and nocturnal fluctuations can be performed in form of diurnal-nocturnal–IOP-profiles (DNP) [11]. Especially nocturnal IOP-measurements, which cannot be provided in an outpatient setting but often show nocturnal IOP-peaks, are of special relevance for the therapy of glaucoma patients [12–13].

Trabeculectomy (TE) augmented with mitomycin C (MMC) remains one of the most popular and effective IOP-lowering surgical techniques for open-angle glaucoma [14]. However, only few studies have examined the effect of TE with MMC on IOP-fluctuations in the course of diurnal-nocturnal-IOP-profiles [15–16]. Therefore, the aim of our study was to analyse the influence of TE with MMC on IOP values and IOP-fluctuation measured during a preoperative and postoperative 48-hour-DNP-IOP-profile. Additionally, the relationship between IOP parameters and other ophthalmological relevant factors, such as lens status, was evaluated.

## Materials and methods

This retrospective study was carried out in accordance with the Declaration of Helsinki. Ethics approval was obtained from the Ethics committee of Rhineland-Palatinate, Germany, which waived the requirement for informed consent of the included subjects. All patients had been treated and evaluated at the Department of Ophthalmology of the University Medical Center of the Johannes Gutenberg University Mainz. We performe about 1500 diurnal-nocturnal IOP profiles per year.

### Patient population

The inclusion criteria of the study were: patients with diagnosis of primary open angle glaucoma (POAG), pseudoexfoliative glaucoma (PEXG), pigment dispersion glaucoma (PG), normal tension glaucoma (NTG) and juvenile glaucoma, which were treated with a primary TE with MMC. Furthermore, these patients must have performed a preoperative (performed a few weeks before TE) and postoperative (performed at least 6 months postoperatively) complete 48-hour diurnal and nocturnal IOP-profile. POAG, PEXG, PG, NTG and juvenile glaucoma were defined according to the guidelines of the European Glaucoma Society [17].

The exclusion criteria were: trabeculectomy or laser trabeculoplasty in the past, trabeculectomy combined with phacoemulsification in the study eye.

The eye with TE surgery was defined as the study eye. Concerning patients who had received a TE in both eyes, the eye with the earlier TE was considered as study eye. The DNP performed before surgery was called “preoperative” or 1. DNP and DNP performed after surgery was called the “postoperative” or 2. DNP. TE with MMC was performed after the first and before the second DNP. In case of multiple preoperative IOP profiles, the most recent DNP before surgery was evaluated. In case of multiple postoperative profiles, the data from the first postoperative 48h DNP-IOP-profile that was performed at least 6 months after TE with MMC was selected.

### 48 h diurnal-nocturnal–IOP-profiles

Each DNP covered 48 hours and included 10 IOP measurements. Measurements were taken at: 8:00 a.m., 2:00 p.m., 6:00 p.m., 9:00 p.m. and 12:00 midnight. IOP measurements were performed at 4 time points on the first day of DNP (day of admission). These were performed at 2:00 p.m., 6:00 p.m., 9:00 p.m. and 12:00 midnight. On the second day of DNP 5 measurements were performed at 8:00 a.m., 2:00 p.m., 6:00 p.m., 9:00 p.m. and 12:00 midnight. On day 3 (day of discharge) a final morning measurement was performed at 8:00 a.m. The daytime measurements (8:00 a.m. to 9:00 p.m.) were performed with Goldmann applanation tonometry (GAT) in a sitting position at the slit lamp. The nighttime measurement (12:00 midnight) was performed with a handheld Perkins applanation tonometry (PAT) in supine position. The measurement of IOP with GAT and PAT required instilling oxybuprocain-HCl/fluorescein-Na (Thilorbin^®^, OmniVision) eye drops in the lower conjunctival cul-de-sac of both eyes. All measurements were performed by experienced ophthalmologists. Mean daytime IOP (IOP_day_) was evaluated using IOP values of the measurements between 8:00 a.m. and 9:00 p.m. Mean nighttime IOP (IOP_night_) corresponded to the IOP measurements taken at midnight. Mean IOP_total_ included all IOP values recorded during one DNP regardless of the time of the measurement. IOP amplitude (ΔIOP) was defined as the difference between the average preoperative and postoperative IOP_total_, IOP_day_ and IOP_night_ and illustrates the pressure-lowering effect of TE with MMC. In order to assess IOP fluctuation, the lowest registered IOP during the DNP was subtracted from the highest IOP of the same DNP. IOP fluctuations were evaluated for all IOP-measurements (fluctuation_total_), daytime- (fluctuation_day_) and nighttime IOPs (fluctuation_night_). Mean IOP values and IOP fluctuations were calculated for both preoperative and postoperative IOP-profiles. Analogous to ΔIOP the Δfluctuation (difference between the average preoperative and postoperative fluctuations) was assessed.

Pre- and postoperative correlation between mean IOP_total_, mean fluctuation_total_, ΔIOP, Δfluctuation and other relevant variables such as: gender, type of glaucoma, lens status, TE of the fellow eye, maximum preoperative IOP (IOP_max_), central corneal thickness (CCT), number of preoperatively applied antiglaucomatous agents, postoperative needling, suturlyses (SL) and 5-fluorouracil (5-FU)-injections were evaluated. IOP_max_ corresponded to the highest documented IOP measured with or without therapy during the whole preoperative period.

### Surgery

According to the hospital´s preoperative standards, all patients underwent a 4-week washout period of all topical IOP lowering medications before TE with MMC. IOP elevation was treated individually with acetazolamide tablets (maximum daily dose: 6 x 250 mg) and potassium supplementation was provided if necessary. Non-preserved dexamethasone eye drops were applied 5 times per day in the study eye for 5 days before surgery. The patients were admitted to the clinic one day prior to surgery. All study eyes underwent fornix-based TE with MMC performed by one of the 3 experienced surgeons. The surgery was either performed in local (retrobulbar) or general anaesthesia. After dissecting the conjunctiva from the limbus, a shallow groove was created directly behind the former conjunctival insertion. Three drops of MMC with a concentration of 0.2 mg/mL were applied to a 7 x 7 mm sponge. The sponge was then placed into the priorly prepared subconjunctival space for 5 minutes. The scleral flap size was 4 x 4 mm and four 10–0 nylon sutures were placed in the scleral flap (2 edge sutures and 2 side sutures stitched tangentially through the scleral flap and the adjacent sclera to allow aqueous humour to flow posteriorly). At the end of the operation, the conjunctiva was sutured into the groove using a running 10.0 nylon suture in a meander-like fashion [18]. This suturing method results in a very tight wound closure.

After TE, all patients stayed on ward as inpatients for about 1 week. Depending on the postoperative bleb assessment, IOP-values and overall scarring tendency SL and/or 5-FU injections were performed.

### Success criteria

The definition of the target IOP after TE and hence the assessment of the surgical success were based on the guidelines of the European Glaucoma Society (EGS) [1]. In the present study a complete success (target IOP without antiglaucoma medication) and qualified success (target IOP with antiglaucoma medication) after TE with MMC was refined by multiple success criteria: a) IOP ≤ 15 mmHg, b) IOP ≤ 18 mmHg or c) a mean IOP reduction > 30% referring to the highest IOP value measured under treatment assessed during the preoperative IOP-DNP-profile.

### Statistical analysis

The collected data was statistically analysed using SPSS 22 (SPSS Inc., Chicago, USA). For descriptive analyses means and standard deviations were calculated for continuous distributed variables and were visualized by box plots. In addition, absolute and relative frequencies were computed for categorical variables and presented as bar charts. Quantitative, normally distributed variables were analysed using the paired or unpaired t-test depending on the type of hypothesis, as well as the Wilcoxon signed-rank test or the Mann–Whitney U test. For analysing the dependence of two continuous variables a Pearson correlation coefficient was used for normally distributed outcome variables, and Spearman's rank correlation coefficient was used for not normally distributed outcome variables. A correlation coefficient was considered to be: very weak if .00-.19; weak if .20-.39; moderate if .40-.59; strong if .60-.79 and very strong if .80–1. Due to multiple testing a Bonferroni correction was performed for the four main hypotheses: IOP-lowering effect of TE on the mean IOP as well as mean IOP-fluctuation. This was applied to both day and night measurements with an adjusted local significance level by p≤.0125. All other analyses are regarded as explorative with p ≤.05.

## Results

92 eyes of 92 patients were enrolled in this study. 62 (67.4%) of patients were female. The mean age at the time of TE with MMC was 64.71 ± 10.5 (range 35–82) years. 50 (54.3%) right eyes and 42 (45.7%) left eyes were included. 63 (68.5%) study eyes had POAG, 19 (20.7%) PEXG, 6 (6.5%) PG, 2 (2.2%) NTG and 2 (2.2%) eyes were suffering from juvenile glaucoma.

Table giving a summary of raw data can be found in the supplementary material (S1 Table).

The mean CCT of all study eyes was 523.6 ± 36 μm (range 444 to 625 μm).

At the time of the “preoperative” DNP 67 (72.8%) study eyes were phakic and 25 (27.2%) pseudophakic. At follow-up 57 (62%) study eyes were phakic. Ten patients had a cataract surgery on the study eye during the time between the first and the second DNP, so that 35 (38.0%) eyes were pseudophakic during the “postoperative” DNP.

“Preoperative” DNP trabeculectomy was performed in 12 (13%) fellow eyes, while „postoperative” DNP trabeculectomy has been performed in 25 (27.2%) of fellow eyes.

The “preoperative” DNP was performed at least 4 weeks and maximally 8 weeks before TE (mean 5.9 ± 1.4 weeks, range: 4–8 weeks). The “postoperative” DNP was performed at least six months after TE (mean 12 ± 5.7 months, range: 6–24 months). During the “preoperative” DNP all study eyes were treated with topical antiglaucomatous agents. Five different groups of antiglaucomatosa (beta-blockers, alpha-agonists, carbonic anhydrase inhibitors, prostaglandins, parasympathomimetics) were used either in monotherapy or in combination. At the timepoint of the 1st DNP on average 3.22 ± 0.94 substances were applied to the study eye. 40 (43.5%) patients received 4.32 (34.8%) 3 and 12 patients (13.0%) received 2 IOP lowering topical substances. A monotherapy was used in 6 (6.5%) patients and 2 (2.2%) patients applied 5 substances. At the timepoint of the 2nd DNP the majority of the patients were not using antiglaucomatous medication on the study eye (75 patients// 81.5%). Only 17 (18.5%) patients required on average 0.41 ± 1.00 (from 0 to 4 substances) antiglaucomatous agents (Table 1).

**Table 1:** Number of applied antiglaucomatous agents in “preoperative” (1st DNP) and “postoperative” diurnal-nocturnal IOP profile (2nd DNP).

Antiglaucomatous agents	Patients at 1st DNP	Patients at 2nd DNP
0	0	75 (81.5%)
1	6 (6.5%)	6 (6.5%)
2	12 (13%)	4 (4.3%)
3	32 (34.8%)	4 (4.3%)
4	40 (43.5%)	3 (3.3%)
5	2 (2.2%)	0%

On average 0.74 ± 0.88 SL (range 0–4) and 3.30 ± 2.61 5-FU injections (range 0–10) were performed during the immediate postoperative period. Eight (8.7%) study eyes were treated with a needling procedure in the period between TE and the 2nd DNP. None of the patients needed re-trabeculectomy in this period.

### IOP values and IOP fluctuation

For the entire patient population mean IOP_max_ (the highest documented IOP which was measured with or without therapy during the whole patient´s glaucoma history) was 29.52 ± 7.86 mmHg.

The maximal mean IOP (under topical antiglaucomatous therapy) in the “preoperative” DNP was 18.83 ± 4.73 mmHg. The maximal values were recorded on day 1 at 00:00. The minimal mean “preoperative” IOP was 15.53 ± 5.31 mmHg and was recorded on day 3 of the 1st DNP at 8:00 a.m.

**Fig 1:**
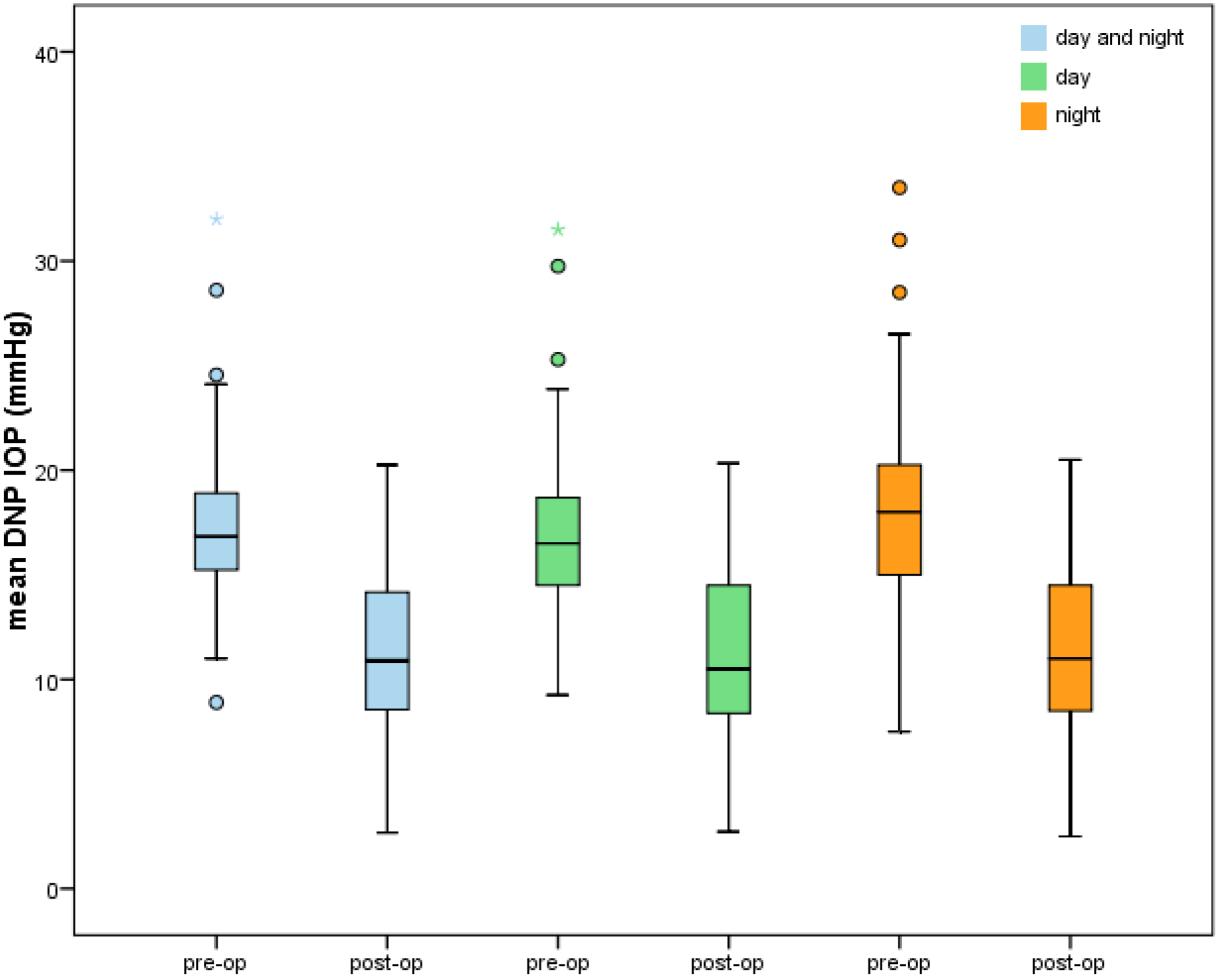
Mean intraocular pressure (IOP) measured during daytime, at night and during the whole first (“pre-op”) and second diurnal-nocturnal IOP (“post-op”) profile [mmHg]. Outliers—values beyond 1.5 x interquartile range—are marked as circles (“out” values) and stars (“far out” values).

**Fig 2:**
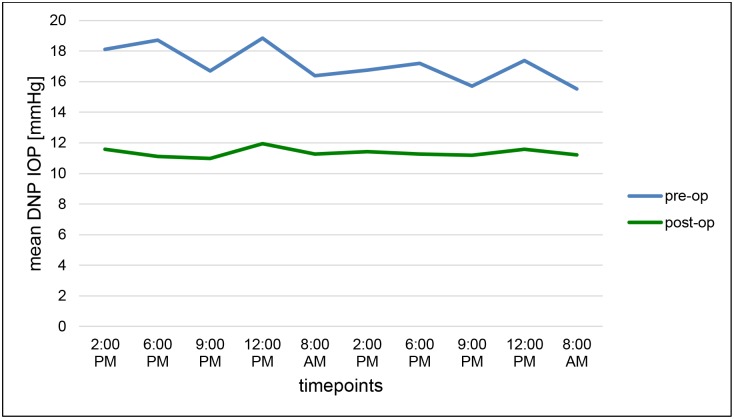
Course of the intraocular pressure values (IOP) at all timepoints in the first (”pre-op”) and second diurnal-nocturnal IOP profile (”post-op”).

TE with MMC led to a remarkable long-time IOP reduction at each timepoint of IOP measurement during the 2nd DNP. Regardless of the measurement time, mean “preoperative” IOP_total_ could be lowered from 17.21 ± 3.60 to a mean “postoperative” IOP_total_ of 11.37 ± 3.69 mmHg (32.4%, p<0.001). Mean IOP_day_ was significantly reduced from 16.94 ± 3.83 to 11.26 ± 3.77 mmHg (31.8%, p<0.001), as well as mean IOP_night_ which was reduced significantly from 18.17 ± 4.26 mmHg to 11.76 ± 3.90 mmHg (32.0%, p <0.001) (Figs 1 and 2).

**Fig 3:**
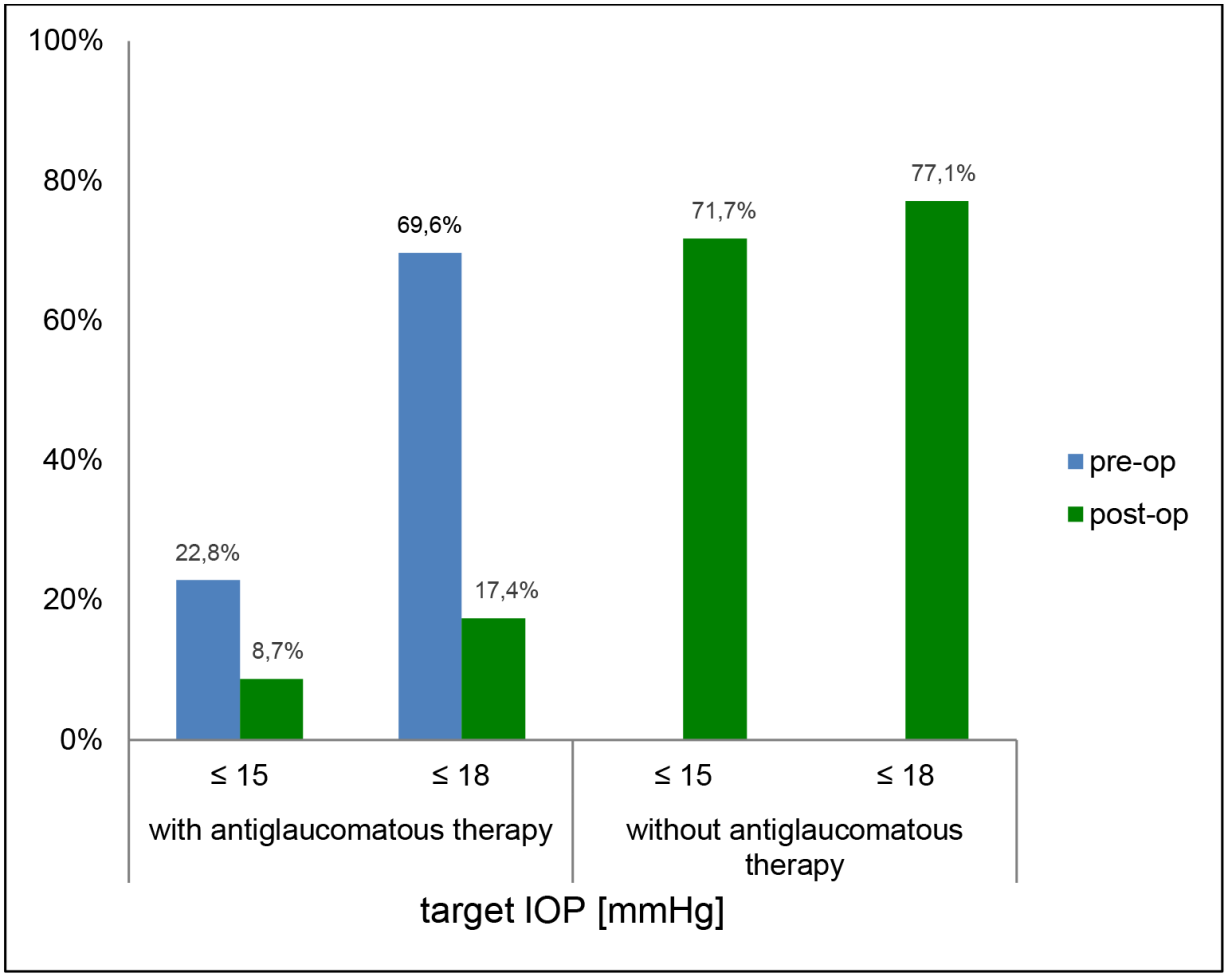
Number of patients who achieved mean IOP_total_ ≤ 15 mmHg and ≤ 18 mmHg with or without antiglaucomatous therapy during the first (“pre-op”) and second diurnal-nocturnal IOP profiles (“post-op”).

**Fig 4:**
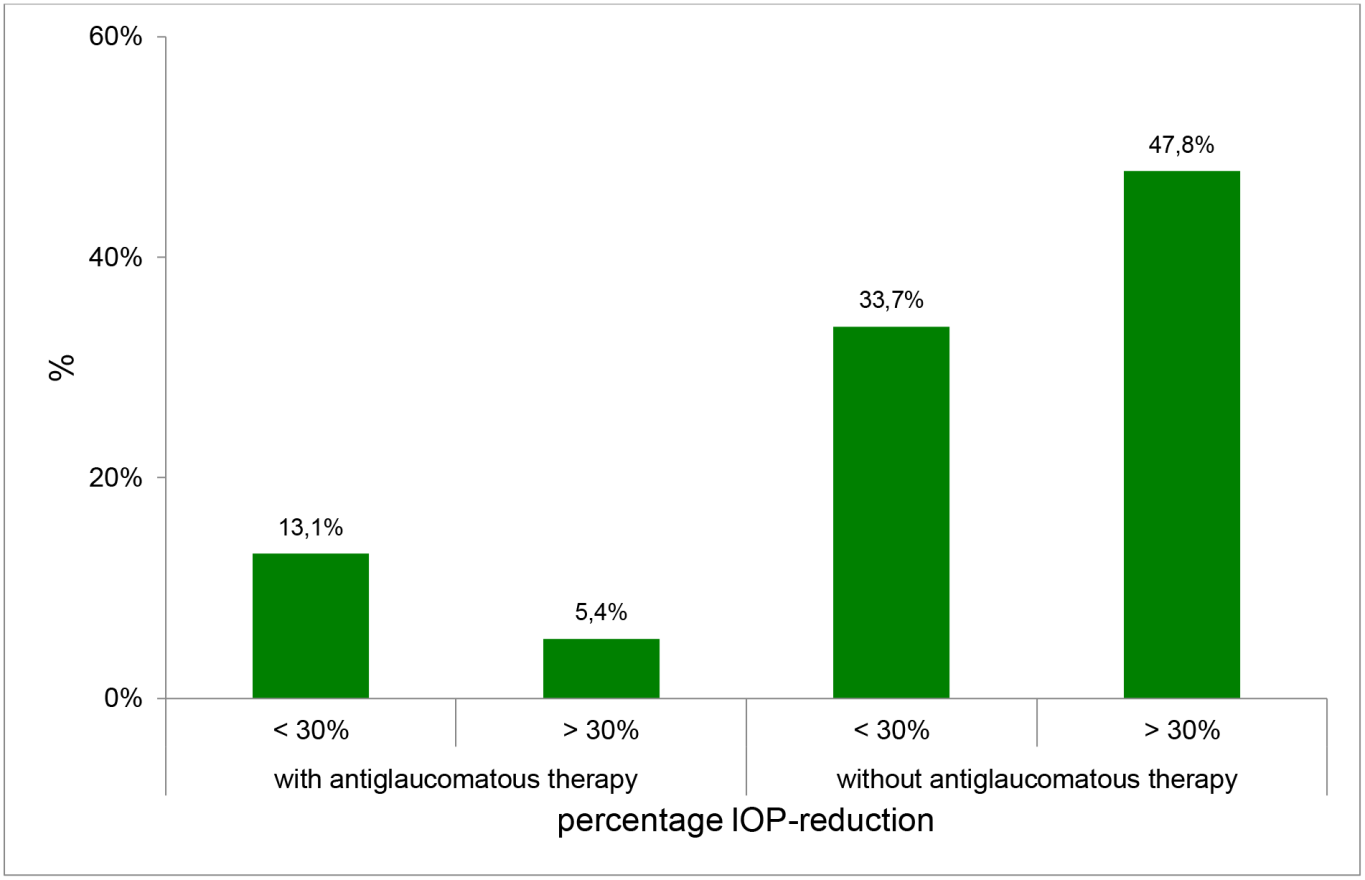
Number of eyes which achieved an IOP-decrease > 30% with or without antiglaucomatous therapy in the second diurnal-nocturnal IOP profile.

At least 6 months after TE with MMC (during the 2. DNP) 71.7% patients achieved an IOP ≤ 15 mmHg; 77.2% showed an IOP ≤ 18 mmHg and 47.8% of the patients had achieved a decrease in IOP > 30% without additional topical antiglaucomatous therapy. 22.8% patients achieved an IOP ≤ 15 mmHg and 69.6% patients achieved IOP ≤ 18 mmHg with topical antiglaucomatous therapy (Figs 3 and 4).

**Fig 5:**
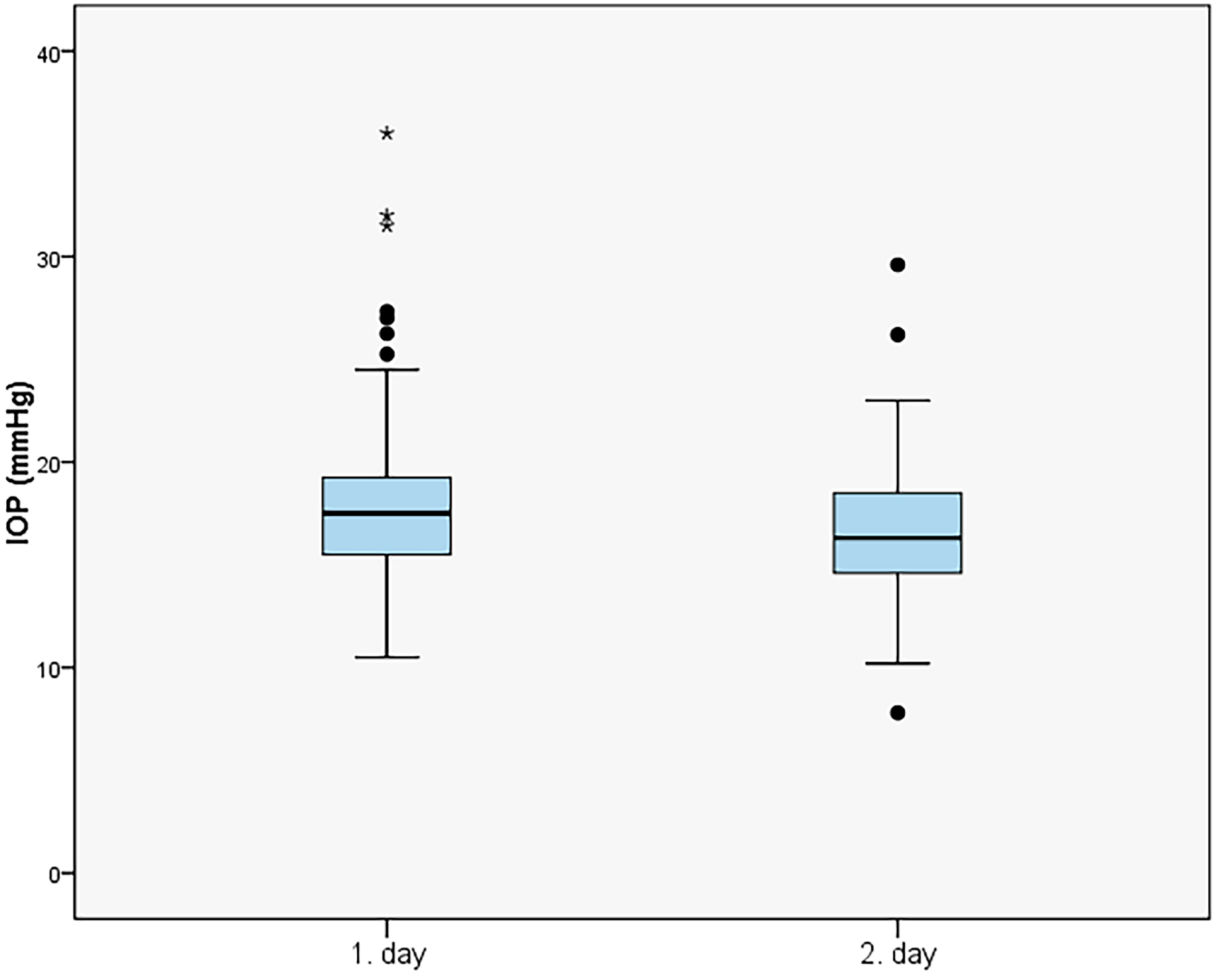
Mean intraocular pressure values (IOP) on the first and second day of the first diurnal-nocturnal IOP profile. Outliers—values beyond 1.5 x interquartile range—are marked as circles (“out” values) and stars (“far out” values).

The measured mean IOP_total_ on the second day of the 1. DNP was at any time considerably lower than on the first day of 1. DNP (16.70 ± 3.37 mmHg vs. 18.13 ± 4.44 mmHg, p<0.001 respectively, Fig 5). During 2. DNP mean IOP_total_ did not show any statistical difference between the first and the second day (11.41 ± 3.67 mmHg vs. 11.33 ± 3.91 mmHg, p = 0.630 respectively).

**Fig 6:**
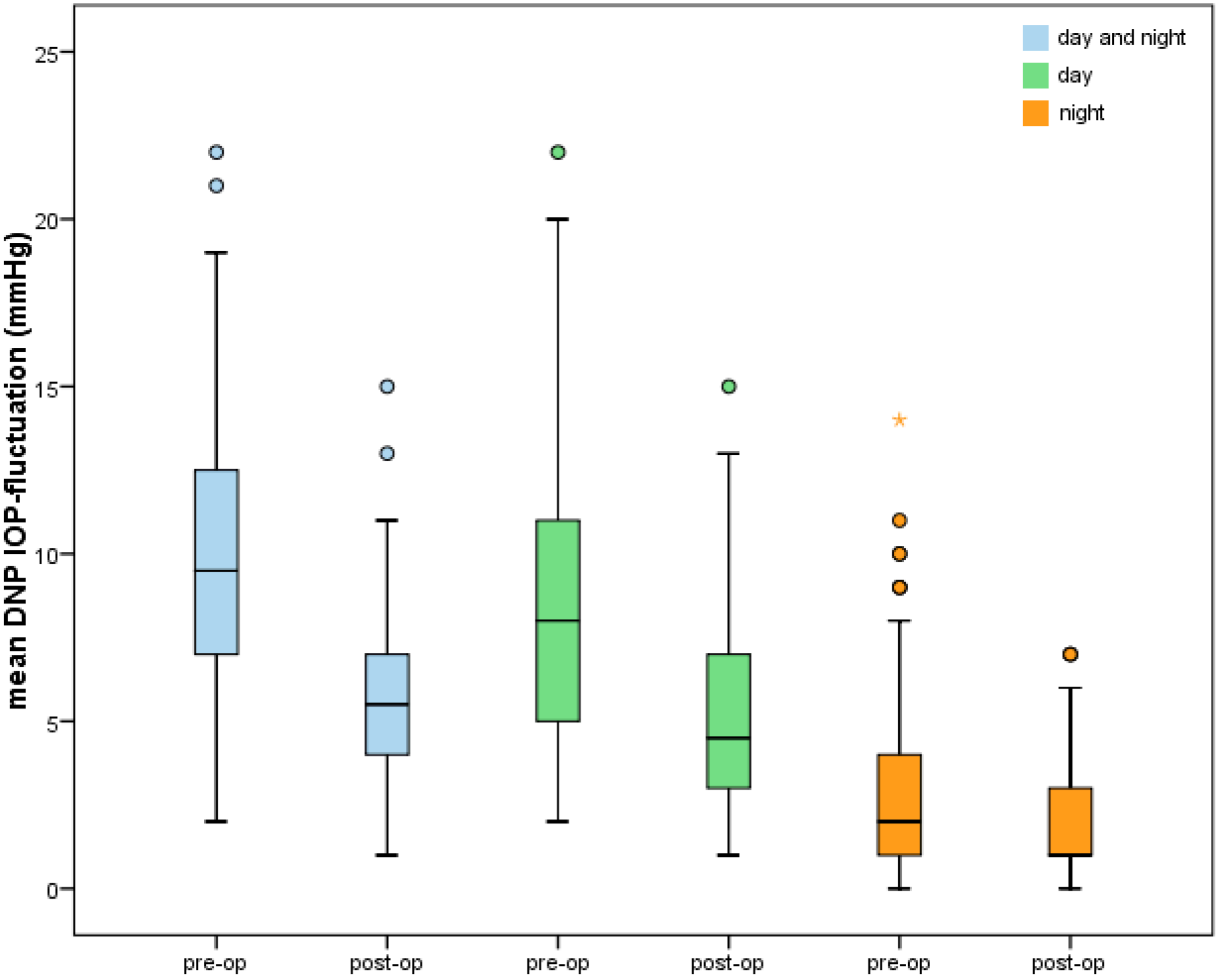
Mean intraocular pressure (IOP) fluctuations measured at daytime, at night and during the whole first (“pre-op”) and second diurnal-nocturnal IOP (“post-op”) profile [mmHg]. Outliers—values beyond 1.5 x interquartile range—are marked as circles (“out” values) and stars (“far out” values).

**Fig 7:**
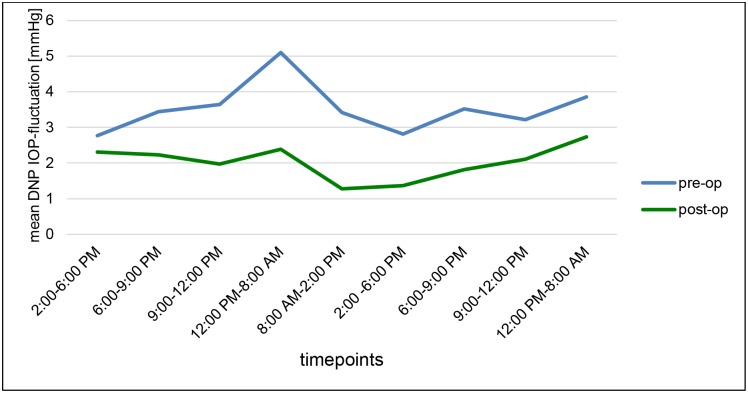
Course of the intraocular pressure (IOP) fluctuations at all timepoints during the first (”pre-op”, blue line) and second diurnal-nocturnal IOP profile (”post-op”, green line) [mmHg].

During the second DNP we could also detect a statistically remarkable reduction in IOP-fluctuation. Regardless of the time of measurement within the 2nd DNP mean fluctuation_total_ was reduced from 10.13 ± 3.89 to 5.78 ± 2.48 mmHg (34.1%, p<0.001). Mean fluctuation_day_ decreased significantly from 8.61 ± 4.19 to 4.92 ± 2.52 mmHg (29.7%, p<0.001) postoperatively, mean fluctuation_night_ showed a reduction from 3.15 ± 2.95 to 1.99 ± 1.82 mmHg (36.9%, p = 0.009) (Figs 6 and 7).

#### Correlation between IOP values/IOP fluctuation and other factors

The only notable correlation of interval scaled variables with mean IOP_total_ and mean fluctuation_total_ or ΔIOP and Δfluctuation was found for IOP_max_ and the number of performed SLs. Preoperative IOP_max_ showed a weak positive correlation with mean IOP_total_ (r = 0.34) and mean fluctuation_total_ (r = 0.28). At least six months after TE with MMC no statistically relevant correlation between IOP_max_ and mean IOP_total_ (r = 0.06) or mean fluctuation_total_ (r = 0.11) was detected. The number of SL was weakly negatively correlated (r = -0.22) with the TE-associated IOP reduction (ΔIOP). The TE with MMC led to a statistical trend of reduction in mean IOP_total_ and mean fluctuation_total_ regardless of gender, form of glaucoma, lens status, history of TE in the fellow eye and postoperative needling. Pseudophakia was associated with a statistically remarkable higher mean fluctuation_total_ after surgery compared to phakic eyes (6.64 ± 2.97 vs. 5.37 ± 2.05 mmHg, p = 0.014). The effect of TE with MMC on mean fluctuation_total_ (Δfluctuation) was less pronounced in pseudophakic than in phakic eyes (2.36 ± 4.03 vs. 5.09 ± 4.22 mmHg, p = 0.006).

## Discussion

The aim of our study was to investigate the influence of TE with MMC on IOP and IOP fluctuations measured by diurnal-nocturnal IOP profiles. To the best of our knowledge this is the first study evaluating 48-hour DNPs in this context. We recorded a statistically significant reduction of both postoperative mean IOP values and postoperative mean IOP fluctuations. More than 70% of the included patients demonstrated a mean diurnal/nocturnal IOP below 15 mmHg without use of additional topical antiglaucomatous therapy during the second DNP. A postoperative decrease above 30% of preoperatively medicated mean IOP was achieved in almost half of the patients without applying additional antiglaucomatous agents. Moreover, the post-TE IOP-curves markedly flattened during day and also nighttime. The postoperative IOP fluctuations were significantly reduced in comparison to preoperative values. This effect was more pronounced at nighttime than at daytime.

Klink *et al*. [15] retrospectively analysed pre- and postoperative 24-hour DNPs of 35 patients after undergoing TE with or without antimetabolites. In comparison to our study showing an IOP-reduction of 31.8%, they were able to demonstrate a higher postoperative IOP reduction of 40.0% during daytime. This is probably due to the higher maximal preoperative IOP-values evaluated by Klink *et al*. (26.5 ± 5.9 mmHg at daytime and 23.4 ± 5.2 mmHg at nighttime under 2.0 ± 1.2 antiglaucomatous agents) in comparison to our study population. However, IOP-reduction of 32% at nighttime correlated in both studies. Most of our study patients had a mean preoperative medicated IOP of less than 21mmHg or even 18 mmHg, but less than a quarter had a medicated IOP of less than 15 mmHg. We defined our postoperative target IOP below 15 mmHg. This restrictive endpoint was achieved in over 80% of the patients.

Only few studies have analysed the influence of filtering surgery on IOP-fluctuations. Klink *et al*. found a postoperative bisection of IOP-fluctuations to the level of 6.1 ± 1.6 mmHg at daytime and 3.9 ± 4.1 mmHg at nighttime. Saiz *et al*. found that both the maximum IOP and the amplitude of the IOP oscillations were significantly reduced one and five years after TE [19]. Wilensky *et al*. included 9 patients performing home tonometry using a Zeimer self-tonometer for at least 3 days before and at least 3 months after filtering surgery [16]. He found a lowering of the mean diurnal IOP from 21.3 ± 1.3 preoperatively to 12.3 ± 1.1 mmHg postoperatively, as well as a reduction of the day-to-day variability from 5.0 ± 0.6 to 2.4 ± 0.5 mmHg after filtering surgery. Unfortunately, the filtering surgery technique was not described in this study. Additionally, there is no information on the amount of used topical antiglaucoma therapy or additional glaucoma surgery.

All patients included in our study underwent primary TE augmented with MMC performed in the same manner. No other glaucoma surgery or laser trabeculoplasy had been performed in the study eye previously. IOP measurements performed beyond the office hours allow a reliable assessment of therapy success or failure in glaucoma patients. Among other things, they allow the assessment of nocturnal IOP-values in supine position, which are known to be frequently higher than daytime IOP-values measured in a sitting position [20]. Whether repeated IOP measurements beyond 24 hours provide relevant additional information, is currently under discussion [21]. When evaluating the IOP measurements registered in non-operated eyes, statistically significant lower mean IOP-values could be found on the second day in comparison to the first day of the performed DNP. This may be due to both psychological and physiological factors. Therefore, we are convinced that 48-hour DNP are superior to 24-hour IOP profiles. About 1500 diurnal-nocturnal 48-hour IOP profiles are performed in the Department of Ophthalmology of the University Medical Center of the Johannes Gutenberg University Mainz per year. They allow us to prove the efficacy of the topical therapy, as well as to justify the necessity of glaucoma surgery.

In his recent review Konstas *et al*. supports the advantages of 24 hour IOP-profiles in glaucoma management [22]. They enhance our understanding of the role of elevated IOP in glaucoma initiation and progression. With regard to our results, IOP oscillations can be more easily recognized by measurements performed throughout a 48-hour period. In contrast to the “preoperative” DNP we could not detect any significant IOP-differences between day 1 and 2 of the DNP during the “postoperative” DNP. This effect confirms the stable long-term IOP-reduction achieved with TE with MMC.

Documentation of IOP_max_—the highest documented IOP, which was measured with or without therapy during the whole patient´s glaucoma history—facilitates assessment of the individual target IOP. In the present study the mean IOP_max_ showed a slight positive correlation with the mean IOP_total_ and mean IOP fluctuation_total_ in the preoperative DNP. We can speculate that patients with higher IOP_max_ benefit from a continuous measurement to a higher degree due to the fact that irregularities of IOP parameters may be detected with a higher precision during DNP. IOP_max_ also showed a slight positive correlation with the IOP-lowering effect of TE with MMC (ΔIOP). Patients with higher IOP_max_ had a larger benefit from the filtrating surgery. The incidence of immediate postoperative SL showed a slightly negative correlation with the TE-associated long-term pressure reduction (ΔIOP). Therefore, frequent SL could indicate inadequate long term filtration. Interestingly, TE in pseudophakic eyes seems to be less successful in comparison to phakic eyes [23]. Pseudophakia was associated with higher IOP fluctuation_total_ after TE with MMC compared to phakic patients. The difference was statistically striking, but clinically small. Additionally, the effect of TE with MMC on Δfluctuation in preoperatively pseudophakic patients was lower than in phakic patients.

The limitation of our study is a lack of further nighttime IOP-measurements. To evaluate the nocturnal IOP fluctuations accurately, repeated IOP measurements at several times during the night would be desirable. Furthermore, a prospective trial with a longer follow-up and better defined time points of postoperative DNPs (e.g. 6, 12, 18, 24 months) and assessment of functional parameters, such as visual field, should be considered.

In conclusion, we showed high efficacy of TE with MMC to lower IOP values and IOP fluctuations at both day- and nighttime for at least six months postoperatively. The remarkable IOP differences between the first and the second day of the DNP in non-operated eyes imply the necessity of IOP profile that last longer than 24 hours.

## Supporting information

S1 Table Raw data_DNP.xlsx. (XLSX)
